# Double-Inlet Single Ventricle with Malposed Great Arteries

**DOI:** 10.5935/abc.20190160

**Published:** 2019-09

**Authors:** Paulo Andrade, Danilo Santos, Magna Moreira, Adail Almeida

**Affiliations:** Hospital UNIMEC - Cardiologia, Vitoria da Conquista, BA - Brazil

**Keywords:** Transposition of great vessels/ surgery, Mitral Valve Insufficiency, Diagnosis, Imaging, Echocardiography, Doppler/ methods, Aged

A 62-year-old man came to the Echocardiography Service with a history of arterial hypertension and systolic murmur in the mitral area. At the subsequent evaluation, the patient reported dyspnea and fatigue on moderate exertion, but without an impact on social life. Peripheral oxygen saturation at rest ranged from 95% to 98%; extremities were warm and perfused, with no signs of peripheral hypoperfusion; cyanosis and digital clubbing were absent.

**Figure 1:**
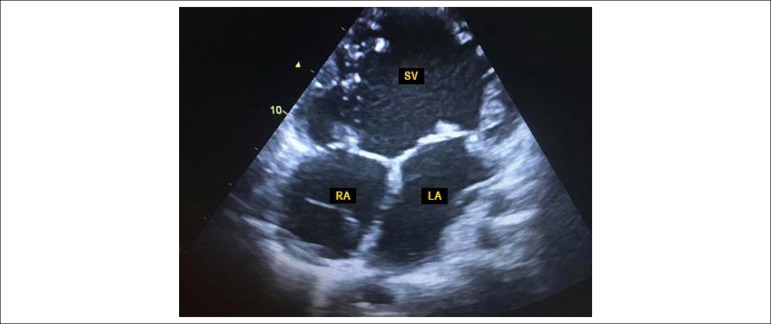
Transthoracic echocardiography: apical view, showing single ventricle and no evidence of recorded interventricular septal tissue. 254x190mm (96x96 DPI).

**Figure 2:**
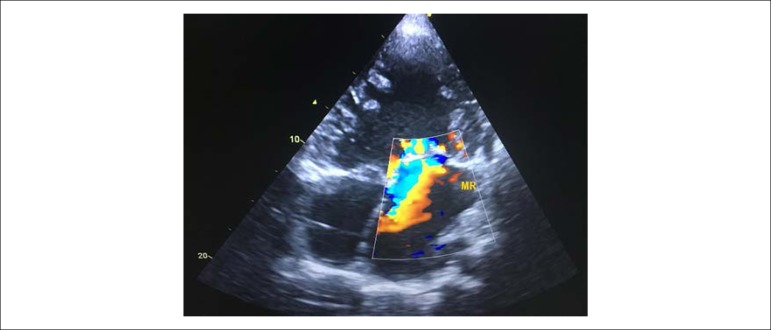
Transthoracic echocardiography: apical view, demonstrating two atrioventricular valves, interatrial septum and mitral regurgitation. 361x270mm (72x72 DPI).

**Figure 3:**
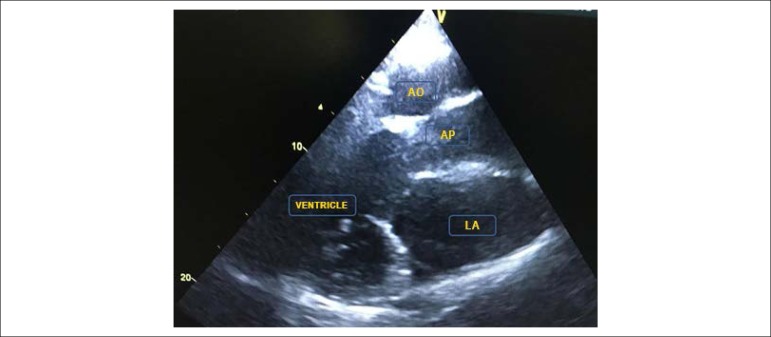
Long axis, parasternal view showing the transposition of the great arteries. 254x190mm (96x96 DPI).

The echocardiogram disclosed a case of levocardia, with the presence of a double-inlet single ventricle with transposition of the great arteries (Figures 1, 2 and 3), with *situs solitus*, enlargement of the atrial chambers associated with significant mitral regurgitation due to annulus dilatation.

**Figure 4:**
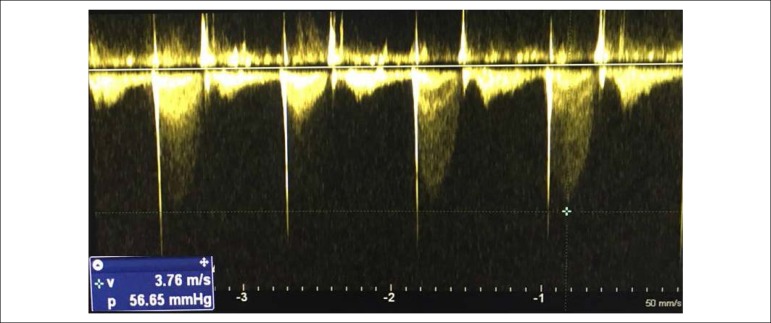
Pulmonary gradient. 254x190 mm (96x6 DPI).

The anatomical preservation of the two atrioventricular valves was observed, as shown in Figure 1. It was not possible to define the type of ventricle from a morphological perspective, but increased dimensions and moderate contractile dysfunction were observed. The presence of pulmonary stenosis with a maximum gradient of 56 mmHg was observed, as depicted in Figure 4.

The single ventricle refers to an uncommon condition that corresponds to 1.5% of congenital heart diseases, in which a single pumping chamber receives the inflow of the two atria,^1,2^ being uncommon in oligo- or asymptomatic elderly individuals, without previous surgical correction. A second rudimentary chamber may be present, but there is no functional entry.^1^ Based on the morphology, location and the trabeculation pattern of the pumping and rudimentary chambers, the heart is referred to as right, left or undetermined univentricular heart,^3^ as in the present report. The most common form of single ventricle is the left ventricular type, where the ventricle connections are variable;^4^ in this case, there was also transposition of the large vessels.

The echocardiography was essential for the diagnosis of double-inlet single ventricle, but it is not always possible to establish the type of ventricle, i.e., whether it is right or left, since it becomes difficult to be certain there is no second rudimentary ventricle. In these cases, magnetic resonance imaging is required for diagnostic complementation.
